# Spectroscopic and Physicochemical Investigations of Azomethines with Triphenylamine Core towards Optoelectronics

**DOI:** 10.3390/ma15207197

**Published:** 2022-10-15

**Authors:** Muhammad Faisal Amin, Paweł Gnida, Sonia Kotowicz, Jan Grzegorz Małecki, Mariola Siwy, Paweł Nitschke, Ewa Schab-Balcerzak

**Affiliations:** 1 Centre of Polymer and Carbon Materials, Polish Academy of Sciences, 34 M. Curie-Skłodowska Str., 41-819 Zabrze, Poland; 2 Institute of Chemistry, University of Silesia, 9 Szkolna Str., 40-007 Katowice, Poland

**Keywords:** triphenylamine, azomethines, photovoltaics, bulk-heterojunction solar cells

## Abstract

Three new azomethines based on triphenylamine with two or three substituents were obtained. Chemical structure and purity were confirmed by ^1^H NMR, FTIR elemental analysis and mass spectroscopy. The investigations were focused on the relationship between chemical structure and properties important for optoelectronic materials. Thus, the studies of thermal, optical and electrochemical properties were carried out based on differential scanning calorimetry, thermogravimetric analysis, electronic absorption, photoluminescence and cyclic voltammetry measurements. The ongoing consideration of experimental results was complemented by theoretical calculations using the density functional theory method. The donor activity of obtained compounds was tested in bulk-heterojuntion photovoltaic cells with structure ITO/PEDOT:PSS/imine:PCBM/Al and ITO/PEDOT:PSS/imine:P3HT:PCBM/Al). The effect of the presence of the amino-thiophene-3,4-dicarboxylic acid diethyl ester groups and various number of hexyloxyphenyl units on imines properties was demonstrated.

## 1. Introduction

Developing organic π-conjugated molecules for optoelectronics is the major research interest since the discovery of polyacetylene [1]. Replacing expensive and non-renewable inorganic materials with cheap, green and reproducible organic materials is the aim of today’s world. The ease of synthesis of organic materials, their low production cost, easy purification and tunable optical and electrochemical properties lend their use in various optoelectronic devices such as light emitting diodes, transistors, lasers and solar cells [2,3,4]. Considering their wide applicability, various synthetic routes have been developed to synthesize these compounds in high yields with great ease and good reproducibility. Out of vast majority of organic π-conjugated materials, azomethines are of considerable importance due to their extremely facile synthesis and easy purification procedures. Unlike C-C bond forming chemistry following Suzuki-, Heck-, or Stille-type couplings [5,6,7] which involve stringent synthesis conditions, incorporating the use expensive transition metal catalysts and later an extensive purification of product, Schiff base condensation chemistry is an attractive alternative to synthesize π-conjugated small organic molecules and polymers. Aldehydes and amines, which are to be condensed together, can tailor the optical and electrochemical properties of synthesized azomethines [8]. Immense research is focused to synthesize organic π-conjugated materials incorporating azomethine moiety (-C = N-) with optoelectronic properties up to the mark which can compete with their expensive counterparts [9,10,11,12,13,14].

Among various azomethines, triphenylamine (TPA) based imines have tremendous importance, particularly in the sector of solar cells. Since 2010, triphenylamine based polyazomethines and small azomethine molecules have been extensively employed either as hole transporting materials or as dyes in various types of solar cells [13]. Conjugation of the core electrons is one of the basic properties of triphenylamines for their use in electronic devices [14]. The propeller shaped structure of triphenylamines core causes π-conjugation by stretching of C-N bond as well as by torsion around carbon nitrogen bond [15,16]. Their non-planar trigonal structure not only prevents undesirable aggregation of the triphenylamine based compounds, but also protects the active layer of solar cells from damaging [17,18]. High ionization potential of 6.608 eV verifies them to be good electron donors [19]. Combinations of such properties as high oxidation potential and good hole transport properties make triphenylamines a promising candidates for organic electronic devices such as solar cells [20]. The HOMO and LUMO levels of triphenylamine core can be adjusted by substituting it with appropriate groups. The advantage of specifically fine tuning the electrochemical properties of triphenylamine has impelled the material chemists to explore it further. Its derivatives bearing the electron withdrawing and electron donating substituents are widely reported in the literature [21,22,23,24].

Bogdanowicz K.A. et al. received the power conversion efficiency (PCE) of 14.4% while using triphenylamine (TPA) based imines as hole transport materials in perovskite solar cells (PSCs) [25]. Korzec M. et al. tested triphenylamine based azomethines as hole transporting materials in PSCs where they received short circuit current density of 7.44 ± 0.11% [mA/cm^2^] and power conversion efficiency (PCE) of 1.1%, which was higher than other non-triphenylamine containing azomethines [26]. The effect of alkoxy chain length on the donating character of azomethines was tested in bulk heterojunction (BHJ) solar cells, and the enhancement of PCE by increasing alkoxy chain length was observed [27]. Damaceanu M.-D. et al. prepared dye sensitized solar cells based upon hexyloxy substituted triphenylamine containing imines and obtained highest PCE of 4.21% [28].

Keeping in mind the potential of triphenylamine based azomethines, herein three different, low cost, solution-processable TPA-based azomethines were synthesized and characterized. It seems that utilization of appropriately substituted amine and aldehyde for condensation results in imines with desirable optoelectronic properties desired in BHJ solar cells. However, the complexity of the phenomena which determine the properties of organic materials creates difficulties in estimating their abilities for applications in various fields of optoelectronics. Therefore, the basic research is very important to determine the influence of various factors on crucial properties determining its optoelectronic response. Thus, the aim of the presented research is focused on investigations of the relationship between such factors of TPA imines as the presence of the amino-thiophene-3,4-dicarboxylic acid diethyl ester groups and various number of hexyloxyphenyl units on thermal, optical and electrochemical properties. Additionally, the electron donor ability of synthesized imines in bulk heterojunction solar cells was exploited. It is expected that the obtained results will extend knowledge of the TPA imines family, which can allow new materials with improved performance to be obtained.

## 2. Experimental Section

### 2.1. Materials

Diethyl-2,5-diamino-3,4-thiophenedicarboxylate was synthesized according to literature [29]; chloroform, chlorobenzene, methanol were purchased from Avantor Performance Materials Poland S.A., Gliwice, Poland; N-Methyl-2-pyrrolidone (NMP), Bis(4-formylphenyl)phenylamine, Tris(4-formylpheny)amine, and 4-Hexyloxyaniline purchased from Sigma-Aldrich Chemical Co. (Merck, Darmstadt, Germany); Poly(3-hexylthiophene) (P3HT, M102, M_w_ = 66,225 g/mol), [6,6]-Phenyl-C61-butyric acid methyl ester (PCBM, M111, >99% wt.), poly(3,4-ethylenedioxythiophene) polystyrene sulfonate dispersion in water (PEDOT:PSS, M124) were purchased from Osilla (Sheffield, UK) have been used without any prior purification.

### 2.2. Measurements

The spectra of ^1^H NMR were recorded on an Avance II 600 MHz Ultra Shield Plus (Bruker) Spectrometer, as a solvent was used CDCl_3_ and DMSO, and TMS as the internal standard. The IR spectra of macromolecular compounds were recorded with a JASCO FT/IR-6700 (JASCO Co. Ltd., Tokyo, Japan) spectrometer, KBr pressed pellets (KBr before use was dried). The spectra were collected over a range of 400–4000 cm^–1^ with a resolution of 4 cm^–1^ and 64 scans for signal accumulation. Mass spectra were recorded on a Finnigan LCQ ion-trap mass spectrometer (Thermo Finnigan, San Jose, CA, USA). The elemental analysis was performed using Vario EL III apparatus (Elementar, Langenselbold, Germany) where helium was the carrier gas. The absorption spectra in the UV–Vis range were recorded in solutions and as thin films on glass substrates using a V-570 UV–Vis–NIR Spectrophotometer (Jasco Inc. Tokio, Japan). The emission and excitation spectra were recorded using a Hitachi F-2500 Spectrometer. Differential scanning calorimetry (DSC) was performed with a TA-DSC 2010 apparatus (TA Instruments, New Castle, DE, USA) under nitrogen using heating/cooling cycles of 20 °C min^−1^ rates. Thermogravimetric analysis was performed on Mettler Toledo TGA STARe system (Warszawa, Poland) with a heating rate of 10 °C∙min^−1^ in a constant stream of nitrogen (20 mL∙min^−1^). Electrochemical measurements (cyclic voltammetry (CV) and differential pulse voltammetry (DPV)) were performed with Eco Chemie Autolab PGSTAT128n potentiostat in a one-compartment cell in dichloromethane (Merck Darmstadt, Germany, for HPLC, 99.8%), with the supporting Bu_4_NPF_6_ (Merck, 99%) electrolyte salt with the concentration 0.1 mol/dm^3^. A platinum wire (diam. 2.0 mm) served as a working electrode, and the platinum coil and silver wire were used as auxiliary and reference electrode, respectively. Experiments were performed at 23 °C ± 1 °C in air atmosphere, and after 5 min argon purging. The measurements were recorded with moderate scan rate equal 0.1 V/s for cyclic voltammetry and 0.05 V/s for differential pulse voltammetry. Potentials were referenced to the stable Fc/Fc^+^ couple with IP = −5.1 eV (ionization potential, E_HOMO_) [30]. E_HOMO_ as ionization potentials and E_LUMO_ as electron affinities are related to HOMO and LUMO energy levels. The morphology and thickness of the surface of thin film, blends and devices in nanoscale were characterized by atomic force microscopy (AFM) using TopoMetrix Explorer device, operating in contact mode, in air, in constant force regime. The photovoltaic parameters were determined by a PV Solutions Solar Simulator (PV Test Solutions, Wrocław, Poland) and a Keithley 2400 (under AM 1.5 G, illumination 100 mW/cm^2^) (Tektronix, Inc., Beaverton, OR, USA).

### 2.3. Synthesis of Triphenylamine Based Azomethines

#### 2.3.1. Synthesis of TPA-DT

Into the flask was placed 660.15 mg (2.5 mmol) of diethyl-2,5-diamino-3,4-thiophenedicarboxylate and then 5 mL of chloroform was added. The mixture was stirred until the precipitate dissolved. In the next step, 164.71 mg (0.5 mmol) of tris(4-formylphenyl)amine was added. The reaction was carried out at room temperature without using any catalyst. The product was precipitated in methanol. The solution with the precipitate was cooled to about 4 °C and left at this temperature overnight. The precipitate was filtered and dried in air. Yield: 86%. FTIR (KBr, υ, cm^−1^): 3424, 3307 (NH_2_ stretch), 3171 (C-H aromatic), 2978 (C-H aliphatic), 1732 (C=O), 1641. (CH=N stretch), 1584 (C=C aromatic) 1272 (C-N stretch). ^1^H-NMR (600-MHz, DMSO, δppm): 7.96 (s, 3H, HC=N), 7.83 (s, 6H, NH_2_), 7.71 (d, 6H, Ar-H), 7.18 (d, 6H, Ar-H), 4.23 (q, 12H, OCH_2_), 1.33 (t, 18H, CH_3_). Calcd. for C_51_H_51_N_7_O_4_S_3_: C, 62.11%, H, 5.50%, N, 9.39%. Found: C, 59.00%, H, 4.887%, N, 9.163%. EI-MS Calcd. for C_51_H_51_N_7_O_4_S_3_, 1050.14. Found 1049.95.

#### 2.3.2. Synthesis of TPA-tHB

166.12 mg (0.5 mmol) of Tris(4-formyl)phenylamine and 300.12 mg (1.5 mmol) of 4-Hexyloxyaniline were placed together in a round bottom flask. The reaction was carried out at 75 °C under an inert atmosphere. Then methanol was added to the obtained product. The prepared solution with the precipitate was cooled to about 4 °C and left at this temperature overnight. The product was then filtered and dissolved in chloroform, then precipitated in methanol. The solution with the precipitate was cooled to about 4 °C and left at this temperature overnight. The precipitate was filtered and dried in air. Yield: 83%, FTIR (KBr, υ, cm^−1^): 3036 (C-H aromatic), 2924, 2855 (C-H aliphatic), 1619 (CH=N stretch), 1597 (C=C aromatic), 1270 (C-N stretch), 1243, 1026 (C-O stretch). ^1^H-NMR (600-MHz, CDCl_3_, δppm): 8.44 (s, 3H, HC=N), 7.81 (d, 6H, CH), 7.22 (d, 6H, CH), 6.95 (d, 12H, CH), 3.98 (t, 6H, OCH_2_), 1.79 (m, 6H, CH_2_), 1.47 (m, 12H, CH_2_), 1.35 (m, 6H, CH_2_), 0.91 (t, 9H, CH_3_). Anal. Calcd. for C_57_H_66_N_4_O_3_: C, 80.06%; H, 7.78%; N, 5.61%. Found: C, 78.36%, H, 7.38%, N, 5.38%. EI-MS Calcd. for C_57_H_66_N_4_O_3_, 855.16. Found 855.60.

#### 2.3.3. Synthesis of TPA-dHB

151.43 mg (0.5 mmol) bis(4-formyl)phenylamine and 201.73 mg (1 mmol) 4-hexyloxyaniline were placed in the flask. To the total, 1 mL of chloroform was added. The reaction was carried out at 50 °C under reflux. The solution with the precipitate was cooled to about 4 °C and left at this temperature overnight. The precipitate was filtered and dried in air. Yield: 30%. FTIR (KBr, υ, cm^−1^): 3034 (C-H aromatic), 2929, 2864 (C-H aliphatic), 1618 (CH = N stretch), 1591 (C=C aromatic), 1283 (C-N stretch), 1244, 1025 (C-O stretch). ^1^H-NMR (600-MHz, CDCl_3_, δppm): 8.40 (s, 2H, HC=N), 7.73 (d, 2H, CH), 7.17 (d, 1H, CH), 6.93 (d, 6H, CH), 3.97 (t, 4H, OCH_2_), 1.79 (m, 4H, CH_2_), 1.47 (m, 8H, CH_2_), 1.35 (m, 4H, CH_2_), 0.91 (t, 6H, CH_3_). Anal. Calcd. for C_40_H_39_N_5_O_8_S_2_: C, 81.07%; H, 7.58%; N, 6.45%. Found: C, 80.61%, H, 7.48%, N, 6.001%. EI-MS Calcd. for C_40_H_39_N_5_O_8_S_2_, 651.92. Found 652.45.

## 3. Results and Discussion

**Figure 1:**
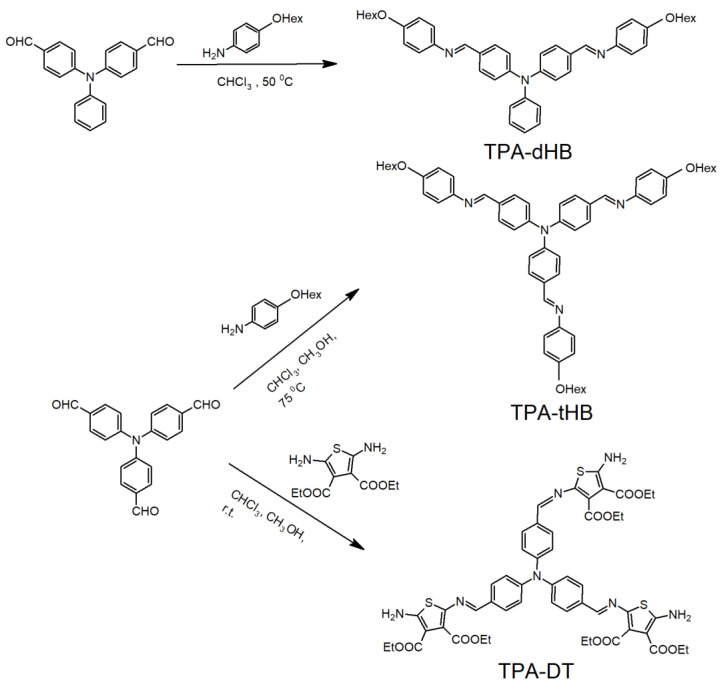
Synthetic route of the azomethines.

Three novel triphenylamine based azomethines were synthesized by the condensation of tris(4-formylphenyl)phenylamine with tiethyl-2,5-diamino-3,4-thiophenedicarboxylate (**TPA-DT**) and 4-hexyloxyaniline (**TPA-tHB**) and by the condensation of bis(4-formylphenyl)phenylamine with 4-hexyloxyaniline (**TPA-dHB**) under mild conditions (Figure 1).

After successful synthesis, as evidenced by NMR, IR, elemental analysis and mass spectrometry, the optical properties of these novel azomethines with triphenylamine (TPA) core, both in solution as well as in thin film forms and blends, were investigated using UV–Vis and photoluminescence spectroscopy, while their electrochemical properties were examined using cyclic (CV) and differential pulse (DPV) voltammetry. Thermal properties were determined using differential scanning calorimetry (DSC) and thermogravimetric analysis (TGA). Differential functional theory (DFT) calculations were performed to estimate HOMO and LUMO energy levels, while atomic force microscopy (AFM) helped to analyze the morphology of the thin films. The optical and electrochemical properties of these compounds have been subsequently considered in terms of chemical structure of the imine, and they were evaluated as donor materials in bulk-heterojunction (BHJ) solar cells.

### 3.1. Structural Characterization

Chemical structures of the azomethines were confirmed by ^1^H-NMR and IR spectroscopy. Furthermore, mass spectrometry and elemental analysis supported the proposed structural formula and revealed good purity of the materials. ^1^H NMR and IR spectra of these azomethines are shown in Figures S1 and S2, respectively. In ^1^H NMR, spectra peak which appeared in the range of 7.90–8.76 ppm confirms the presence of imine (-HC = N-) bond in all three new imines. No peak observed in the aldehyde region for any of the imines shows the completion of condensation reaction. Furthermore, no peak of amine protons (-NH_2_) appeared in the spectra of **TPA-tHB** and **TPA-dHB,** which confirms the condensation between the reactants to form azomethine moiety. Presence of electronegative oxygen atom in the neighborhood of methylene protons (R-OCH_2_-) down fielded their peak to around 3.97 ppm, and also confirms the presence of ether moiety in both of these dyes. The appearance of a quartet at 4.26 ppm confirms the presence of ester moiety in **TPA-DT**. Peak for amine protons at 7.83 ppm for **TPA-DT** further substantiate the proposed structural formula.

FTIR analysis showed a characteristics peak for imine (C=N) in the range of 1615–1645 cm^−1^ for all the synthesized azomethines. C-N stretching band also appeared in the characteristic region. A band at 1732 cm^−1^ is the characteristics for carbonyl double bond (C=O) in FTIR spectra of **TPA-DT**. Peaks at 3420 cm^−1^ and 3301 cm^−1^ confirms the presence of amino group (-NH_2_) in **TPA-DT.** Aromatic and aliphatic C-H stretching bands appeared at 3164 cm^−1^ and 2978 cm^−1^, respectively. C-O stretching band appeared at 1243 cm^−1^ and 1026 cm^−1^. For **TPA-dHB**, peaks for C-O appeared at 1244 cm^−1^and 1025 cm^−1^ while O-H peak is also absent to further assure the presence of ether moiety in the product.

### 3.2. Thermal Properties

DSC and TGA measurements were carried out in in a nitrogen atmosphere and the obtained thermal data are summarized in Table 1, while DSC and TGA thermograms are shown in Figures S3 and S4, respectively.

A sharp endothermic peak in DSC thermograms was observed during first heating scan representing melting temperature (T_m_) of the compounds. First heating scan led to isotropic liquid, which, when rapidly cooled, formed a glassy state in the case of **TPA-DT** and upon heating in the second run, the glass transition temperature (T_g_) was observed. Neither crystallization nor melting temperature was observed during the second heating scan, indicating formation of stable amorphous stare. This same behavior was seen in the case of TPA substituted with two amino-thiophene-3,4-dicarboxylic acid diethyl ester groups denoted as AzDT-3 in our former work [31]. Compared with AzDT-3 the melting temperature and the glass transition temperature of the **TPA-DT** were lower at about 90 and 47 °C, respectively [31]. It can be seen that the introduction of hexyloxyphenyl units to imine (**TPA-tHB** and **TPA-dHB**) significantly lowered T_m_ compared to **TPA-DT** with thiophene structure. However, the opposite effect of such substituents on thermal stability is seen. Thermal stability was determined by temperatures of 5 and 10% (T_5_ and T_10_) weight loss and temperature of maximum degradation rate (T_max_). The compound with three hexyloxyphenyl substituents (**TPA-tHB**) showed the highest thermal stability due to the highest temperature of decomposition beginning (T_5_ = 393 °C). It is worth noting that azomethine with trisubstituted core showed better thermal properties than disubstituted derivative. This kind of substituent also affects the process of thermal decomposition, which proceeds in two or three stages for imines with hexyloxyphenyl (**TPA-tHB** and **TPA-dHB)** and thiophene (**TPA-DT**) derivative, respectively.

### 3.3. Redox Properties

**Figure 2:**
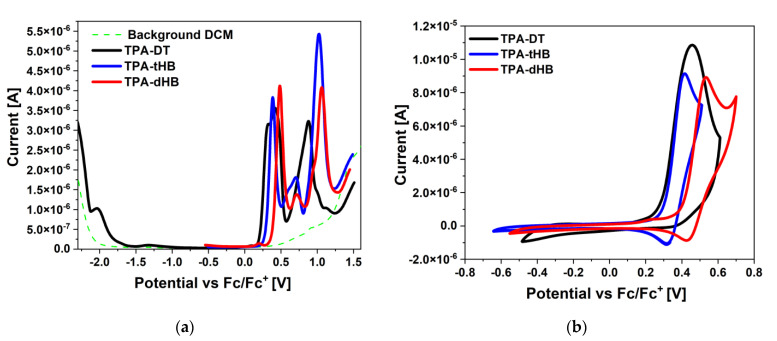
(**a**) DPV and (**b**) CV scans during reduction and oxidation processes (0.1 mol/dm^3^ Bu_4_NPF_6_ in CH_2_Cl_2_ with Pt).

The electrochemical investigations using CV and DPV in the positive and in the negative potential range were performed in dichloromethane (CH_2_Cl_2_) solution with platinum electrode (Pt) as a working electrode. Based on the onset oxidation and reduction potentials the E_HOMO_ and E_LUMO_ were calculated (E_HOMO_ as ionization potentials and E_LUMO_ as electron affinities). The voltammograms are presented in Figure 2 and electrochemical data collected in Table 2.

Presented azomethines were electrochemically active and exhibited two (**TPA-DT**) or three (**TPA-tHB**, **TPA-dHB**) oxidation processes associated with the presence of the electron-donating elements in the molecule (cf. Table 2 and Figure S4 in the Supplementary Materials). The one irreversible reduction process was seen for molecule **TPA-DT** with thiophene rings. The reduction process in the thiophenoazomethines may be related to the reduction in the imine bond and the thiophene ring [27,32]. In the case of azomethine with a triphenylamine core and a two amino-thiophene-3,4-dicarboxylic acid diethyl ester groups, reported in our previous work, two reduction processes were seen (E_red_^1^ = −1.78 V and E_red_^2^ = −2.23 V) [33]. The lack of the second reduction process was caused by the presence of an additional imine bond and a thiophene ring. It is possible that the imine bond reduction process takes place at a greater potential in this case, and the lack of its registration is related to the electrochemical window of the used solvent. The presence of a three amino-thiophene-3,4-dicarboxylic acid diethyl ester substituted hindered the reduction process, which influenced the position of E_LUMO_ and thus the value of the energy band gap. Compounds **TPA-tHB** and **TPA-dHB** did not show the reduction process in a given electrochemical window.

The three oxidation processes were noticed for **TPA-tHB** and **TPA-dHB**, and the presence of an additional ring-aliphatic system facilitated the first oxidation process (E_ox_^1^ = 0.42 V for **TPA-tBH** and E_ox_^1^ = 0.53 V for **TPA-dBH** by CV method). A *quasi*-reversible (∆E = 100 mV) first oxidation process (up to a potential of 0.55 V) was also observed for both compounds. Above the potential 0.55 V, an irreversible second oxidation process was seen (Figure S4). It was also noticeable that an additional hexyloxyphenyl unit had a minor influence on the subsequent oxidation processes. The disappearance of signals from the first and third oxidation processes during scanning at higher potentials was noticed. The imine **TPA-DT** showed similar behavior, and a shift of the second oxidation peak towards higher potential values was noted during the next oxidation scans. In the case of azomethine **TPA-dHB**, polymerization was attempted due to the presence of an unsubstituted ring on the TPA core and the dimerization of the formed cation can occur [31,34]. No potential build-up during subsequent scans, thus it was not possible to obtain an electropolymerization product. This trial was also performed for the compound with TPA core and a two amino-thiophene-3,4-dicarboxylic acid diethyl ester groups presented in our earlier publication [33]. The E_HOMO_ and E_LUMO_ were calculated for **TPA-DT** compound at −3.26 eV and −5.36 eV (E_g_ = 2.10 eV) with the cyclic voltammetry method based on the onset potentials. Its analogue (without one amino-thiophene-3,4-dicarboxylic acid diethyl ester group) exhibited E_HOMO_ and E_LUMO_ at −3.60 eV and −5.24 eV (E_g_ = 1.64 eV) [33]. The obtained value of the energy band gap was lower for the molecule with a two amino-thiophene-3,4-dicarboxylic acid diethyl ester groups, and the oxidation process took place at a lower potential than for **TPA-DT**; the opposite behavior was seen for compounds **TPA-dHB** and **TPA-tHB**. The tested compounds showed potential as p-type materials [35,36].

### 3.4. DFT Calculations

Quantum calculations were carried out using the Gaussian09 program and the calculation details are given in Supplementary Information. Molecular geometry of the singlet ground state of the compounds was optimized in the gas phase on the B3PW91/6–31 g(d,p) level of theory augmented with GD3BJ dispersion correction model. For the compounds a frequency calculation was carried out, verifying that the optimized molecular structure corresponds to energy minimum, thus only positive frequencies were expected. Such calculations were carried out for analysis of the HOMO, LUMO energy levels and UV–vis and photoluminescence data. Optimized geometries and contours of HOMO and LUMO of the compound molecules are presented in Figures S5 and S6 in Supplementary Materials. Comparing the energies of HOMOs and LUMOs determined based on the electrochemical data (Table 2) with theoretically calculated values, it can be noticed that the calculated HOMO energies differ by a maximum of only about 0.3 eV from the experimental values determined from electrochemical measurements. The virtual orbitals are generally more difficult to describe theoretically than the occupied ones. Thus, the errors in the LUMO energy values are usually significantly larger with the calculated LUMO values being much higher in energy than determined experimentally for **TPA-DT**. The average discrepancy between the experimental and the calculated values amounts to about 1 eV (**TPA-DT**). On the other hand, the calculated values of the HOMO and LUMO energies were used only for consistency with geometry optimization. For a more detailed description of the molecular orbitals, the contribution of molecule parts, i.e., triphenylamine, imine (–HC=N–) and substituent fragments (R = amino-thiophene-3,4-dicarboxylic acid diethyl ester and hexyloxyphenyl) to a molecular orbital was calculated. The obtained DOS diagrams are presented in Figure S7 and composition of selected molecular orbitals in ground state are gathered in Table S1. Electronic structures of the **TPA-dHB** and **TPA-tHB** are very similar and HOMO are localized mainly on the triphenylamine with hexyloxyphenyl fragments, while LUMO comprises antibonding orbitals of central NPh_3_ and imine linker. In the **TPA-DT** compound HOMO is localized on amino-thiophene-3,4-dicarboxylic acid diethyl ester unit with NPh_3_, and LUMO includes the π–antibonding orbitals of conjugate bonds over the molecule (Figures S6 and S7 and Table S1 in Supplementary Materials).

Table S3 summarizes calculated dipole moments of the azomethine molecules in solvents. The dipole moment **TPA-tHB** is lower by about 1 D compared to the disubstituted **TPA-dHB** analog. **TPA-DT** shows the highest dipole moment among the studied molecules, which is related to the presence of amino and ester groups in the thiophene substituent. Dipole moments in excited states of the molecules are similar to the values in ground states (maximum difference does not exceed 0.5 D), which explains the lack of solvatochromism in the absorption electronic spectra in solvents with different polarity.

### 3.5. Photophysical Properties

**Figure 3:**
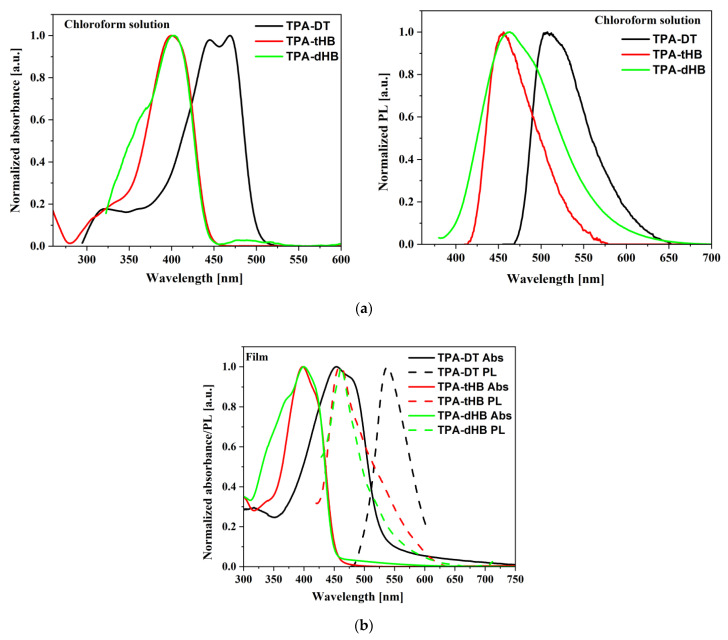
UV–vis and PL spectra of azomethines in chloroform (**a**), and in film (**b**).

**Figure 4:**
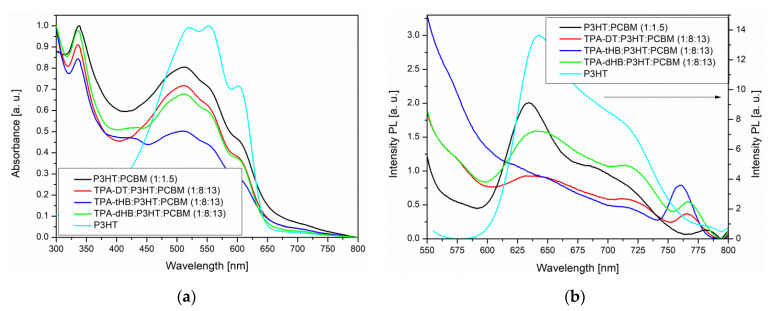
UV–Vis (**a**) and PL (**b**) spectra of imines in blend together with spectra of P3HT and P3HT:PCBM.

Photophysical properties of the synthesized azomethines were investigated by performing UV–Vis absorption and fluorescence emission spectroscopy, both in solution (chloroform (CHCl_3_), chlorobenzene (C_6_H_5_Cl) and dichloromethane (CH_2_Cl_2_)) and in the solid state as thin film obtained from a neat imine and in two types of blends; one consists of azomethine as donor and [6,6]-phenyl-C₆₁-butyric acid methyl ester (PC_61_BM) as acceptor and the second type bearing three components azomethine, poly(3-hexylthiophene-2,5-diyl) (P3HT) as additional donor and PC_61_BM. The UV–vis and photoluminescence (PL) data are summarized in Table 3 and spectra are shown in Figure 3 and Figure 4, Figures S9 and S10.

The imines showed intense absorption band in the range of 300 to 450 or 500 nm, attributed to the intramolecular charge transfer (ICT) which possibly resulted from imine functionality and aromatic core interactions [37]. The absorption at the higher energy range is due to π-π* transitions within the aromatic rings (Figure 3a). Maximum of absorption band (λ_max_) of **TPA-DT** is significantly bathochromically shifted compared to the other imines due to thiophene units. Similar positions of the λ_max_ were also registered for compound with a triphenylamine core and a two amino-thiophene-3,4-dicarboxylic acid diethyl ester groups; however, larger differences were observed for the molar absorption coefficients (higher values for **TPA-DT**) [31]. No noticeable effect of solvent polarity was observed on λ_max_ position of ICT band of the azomethines due to similar dipole moments value in excited and in ground states of the molecules as was found (cf. Section 3.4). The absorption range of the azomethines was red shifted in the case of thin films, as compared to their solutions. It can be a consequence of microstructural changes in solid state compared to solution and the molecules can be more planar in thin films, thus may increase the π-conjugation [38]. The addition of azomethines to mixture PCBM with P3HT did not affect their absorption window (Figure 4).

**Figure 5:**
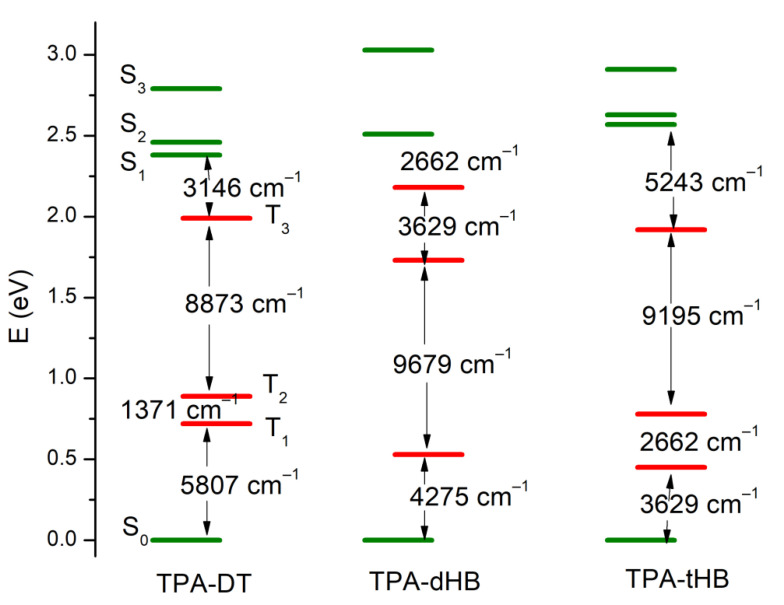
Low-lying energy states in TPA molecules (green lines—the singlet excited states and red lines—triplet excited states).

The PL spectra of the synthesized imines showed emission band with maximum (λ_em_) located at above 500 and 450 nm in the case of compounds with thiophene units (**TPA-DT**) and with hexyloxyphenyl structure, respectively (cf. Table 3). No significant shift in the position of the emission band was observed by the change in solvent polarity. However, the intensity of PL was very weak and the photoluminescence quantum yield (Φ) was in the rage of 0.06 to 2.22%. To explain emission ability of the azomethines the TD-DFT method was used, and the optimization of singlet and triplet excited states were carried out in chloroform. The calculated energy differences between the ground and the first singlet excited state correspond well with the experimental values of emission maxima (cf. Table S4 in Supplementary information). Energy differences in the S_1_ and T_3_ triplet excited states are relatively small (cf. Figure 5 and Table S4), which indicates the possibility of deactivation as a result of internal energy conversion (ISC).

On the other hand, intramolecular photoinduced electron transfer (PET) mechanism may participate in the deactivation of the singlet excited state. In the S_1_ state of **TPA-dHB** and **TPA-tHB** the π-conjugation of the triphenylamine and substituent phenyl ring increases due to changes in molecule geometry (cf. Table S2). The angles between the planes of the rings decrease, which causes a stronger coupling in the N-Ph–HC = N–Ph_subst_ system. On the other hand, in **TPA-DT** in the S_1_ state, the thiophene and phenyl planes are mutually more twisted than in the ground state, which hinders the PET process.

The azomethines were emissive in solid state as film and in the case of **TPA-dHB** and **TPA-tHB** PL quantum yield in film was higher compared to solution. The opposite behavior was seen in the case of **TPA-DT**, where the PL quantum yield in film was lower than in solutions. The observed higher PL quenching in TPA-DT film than the others may result from the presence of heavy atoms like sulfur, which may limit radiation processes [39] and/or formation of aggregates [37]. The azomethine with TPA core substituted with two amino-thiophene-3,4-dicarboxylic acid diethyl ester groups in films obtained from the chloroform and chlorobenzene solutions were non emissive [31]. The PL of imine blends with the same composition as was further used as active layer in photovoltaic cells was examined. For PV devices, the presence of an emission phenomenon is undesirable. To determine the emission quenching properties, a reference blend consisting of P3HT:PCBM (1:1.5) and ternary blends containing the tested compounds **imine**:P3HT:PCBM (1:8:13) was prepared. Hence, the materials forming the active layer should be characterized by a wide absorption band (donor) and the lowest possible emission intensity. As shown in Figure 4a, a P3HT often used as a donor has a wide absorption range from 400 to 650 nm range. However, it can be seen that it also exhibits emission properties. In BHJ solar cells, the active layer is a mixture of donor and acceptor, which should show low emission, and indeed PL of the P3HT:PCBM blend is weaker compared to a neat P3HT (Figure 4b). It is worth noting that a small addition of **TPA-DT** or **TPA-tHB** compounds further lowers the emission of the blends relative to the reference one. Moreover, the addition of **TPA-dHB** is less likely to cause emissions quenching than the other tested imines. It can therefore be concluded that the tested azomethines cause PL quenching, which is desirable for the PV devices. The emission quenching is caused by photoinduced charge separation between electron donating and electron accepting molecules. More efficient emissions extinction could suggest more efficient extinction separation charge transport and separation [40,41]. The addition of **TPA-DT** resulted in a more efficient quenching of PL, which could suggest that this would be the most promising for PV cells.

**Figure 6:**
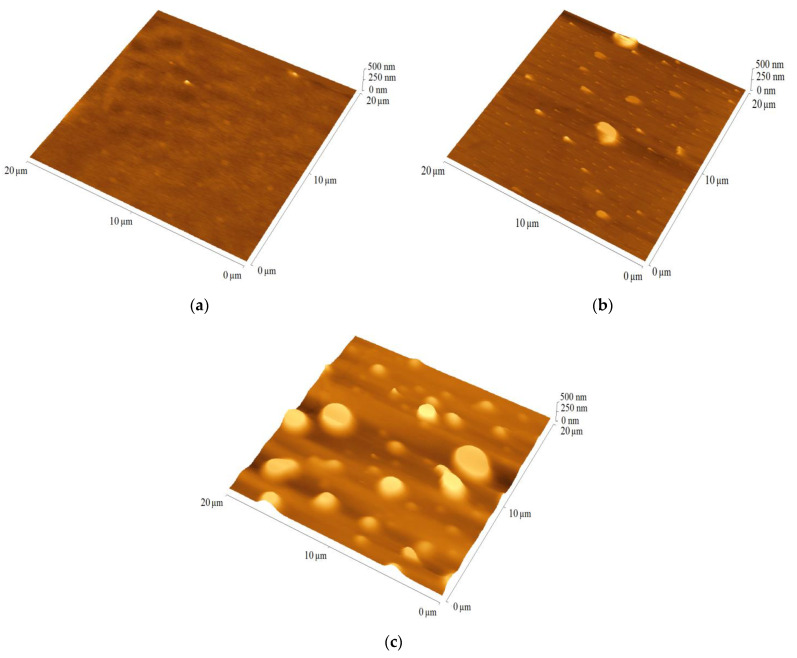
The AFM images of blends (**a**) P3HT:PCBM, (**b**) **TPA-DT**:PCBM and (**c**) **TPA-DT**:P3HT:PCBM.

For PV application important parameters are active layer quality and thickness, which were analyzed using AFM. The quality of the film is indicated by the root-mean-square (RMS) parameter. The films prepared from **TPA-DT, TPA-tHB** and **TPA-dHB** showed thickness ranged from 150 (**TPA-DT)** to 200 nm (**TPA-dHB),** and RMS about 100 to 180 nm. The imines exhibited moderate layer-forming ability. The reference binary blend (P3HT:PCBM) had a thickness of 85 nm and a roughness of 10 nm. The binary blends contained the imines and PCBM were characterized by similar thickness in the range of 40 to 50 nm, with RMS values ranging from 5 to 15 nm. The blends with **TPA-dHB** were the thickest and roughest compared to blends containing **TPA-DT**. The thicknesses of the ternary blends were similar to the thickness of the reference blends at around 80 nm, except **TPA-dHB**:P3HT:PCBM with thickness around 70 nm, while its RMS around 40 nm was higher. Figure 6 shows selected AFM micrograms of blends containing the azomethines.

### 3.6. Photovoltaic Tests

**Figure 7:**
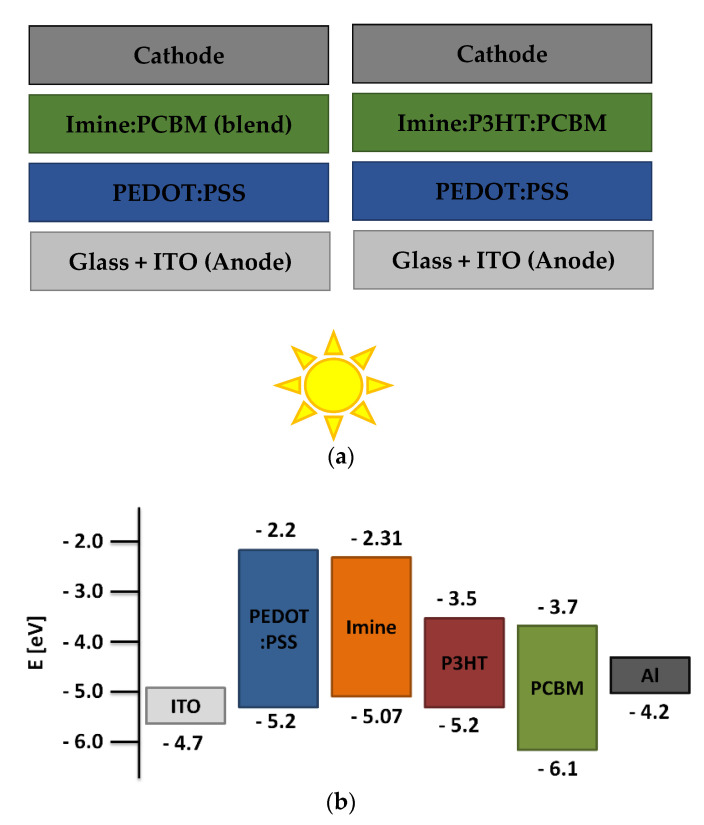
The structures’ scheme of prepared BHJ devices (**a**) and energy diagram with TPA-DT (**b**).

The donor ability of the studied azomethines was tested in the active layer of BHJ photovoltaic cells. A series of devices with two types of structures were prepared. Like the blends previously studied, the active layers in the devices consisted of two or three components. Devices with two- and three-component layers with the structure ITO/PEDOT:PSS/imine:PCBM/Al and ITO/PEDOT:PSS/imine:P3HT:PCBM/Al were fabricated. The structures of prepared solar cells and energy diagram are shown in Figure 7. The active layer thickness (*d*) and roughness were also determined for the devices. The obtained photovoltaic parameters of the devices based on current-voltage measurements are collected in Table 4.

Analyzing the thicknesses of the active layers, it can be seen that those consisting only of imine and acceptor had a lower *d* compared to active layers containing additionally P3HT. The active layer thicknesses of the two-component devices were in the range of 40–55 nm, while for the three-component solar cells the thicknesses were in the range of 80–85 nm. In addition, the thicknesses of the other layers were known, which were for glass (1.1 mm), ITO (100 nm), PEDOT:PSS (40 nm) and Al (100 nm), respectively. Considering the RMS values (5–7 nm) of the surfaces, it can be concluded that they were quite planar with fairly good quality. The surface roughness of the active layers was not significantly affected by the addition of P3HT. The open circuit voltage (V_OC_), short-circuit current (J_SC_), fill factor (FF) and power conversion efficiency (PCE) are the basic parameters defining a photovoltaic cell and depend, respectively, on the matching energy levels of the device components, the absorption properties of the active layers and their quality, and the resistances between the individual layers. Binary blends containing only azomethines as donor materials showed relatively low photovoltaic parameters, as compared to the ternary blends. The highest PCE value was recorded for a device whose active layer consisted of **TPA-DT** and PCBM (0.18 ± 0.02%). Addition of second donor increased the thickness of the active layer and hence caused an increase in the absorption window as well as intensity of absorption of light. This widened absorption window, in turn, elevated the short circuit current density (J_SC_) of the devices containing ternary blends. Again, the highest efficiency (1.05 ± 0.05%) and, noteworthy, a high J_sc_ value (8.20 ± 0.13 mAcm^−2^) was exhibited by the device containing **TPA-DT** in the active layer. The photocurrent-density voltage curves of tested devices are shown in Fig. S11. A device containing an active layer consisting of P3HT:PCBM was prepared as a reference cell. The following PV parameter values were recorded for this solar cell: 488 mV (V_oc_), 11.86 mA cm^−2^ (J_sc_), 0.36 (FF), and 2.07% (PCE). The reference cell exhibited better PV performance, probably due to the lack of optimization of the component’s ratio (imine:P3HT:PCBM), thickness and morphology of the active layer. It was found that a different weight ratio of P3HT to PCBM, as well as additional component concentration [42], has an impact on various properties of active layers, for example, on electron and hole mobility [43], the charge generation [44] and series and shunt resistances [45], which finally impact on PV parameters of devices [46]. In the presented research only one weight ratio of components (1:8:13) was applied and the optimization of cell preparation has not been carried out.

In recent years, research has been conducted into the use of azomethines as an additive to the active layer in PV cells, as evidenced by published work [47]. Polyazomethines are also used as donors; however, BHJ solar cells generally show low efficiencies and the processing of these compounds is organic due to their inferior solubility [48]. However, enhanced power conversion efficiency in bulk heterojunction solar cell based on new polyazomethine with vinylene moieties and [6,6]-phenyl C61 butyric acid methyl ester by adding 10-camphorsulfonic acid was reported [27]. Considering the PV reported results, in which azomethines were used as additives to the active layers in BHJ PV cells, efficiency of 1.05% is low but comparable to the others imines presented as in the literature [21,24,49].

## 4. Conclusions

Three processable azomethines with triphenylamine core substituted with three amino-thiophene-3,4-dicarboxylic acid diethyl ester groups (**TPA-DT)** and two or three hexyloxyphenyl units (**TPA-dHB** and **TPA-tHB)** were synthesized and characterized considering the impact of its structure on selected properties. It can be concluded that:

(a) Substitution of TPA with amino-thiophene-3,4-dicarboxylic acid diethyl ester let to obtained thermally induced amorphous material with high T_g_, and on the other hand it resulted in a decrease in thermal stability compared to azomethine with hexyloxyphenyl structures;
(b) The imine with three hexyloxyphenyl units undergoes oxidation slightly easier, but in the case of azomethines with such substituent reduction was not observed;
(c) Replacement of hexyloxyphenyl groups with amino-thiophene-3,4-dicarboxylic acid diethyl ester units leading to the wide absorption window;
(d) Addition of the synthesized imines to the P3HT:PCBM blend caused emission quenching, thus rationalizing testing them as donors in BHJ solar cells;
(e) The best donor activity showed imine with thiophene rings, and the devices based on its blend with P3HT and PCBM showed the highest J_SC_ of 8.20 mAcm^−2^, which results in the best of PCE.

Summarizing the results it can be concluded that the most perspective considering its thermal and optical properties and donor activity is imine with amino-thiophene-3,4-dicarboxylic acid diethyl ester units.

## Figures and Tables

**Table 1:** Thermal properties of the obtained azomethines.

Code	DSC		TGA		
	T_m_ [°C]	T_g_ [°C]	T_5_ [°C]	T_10_ [°C]	T_max_ [°C]
**TPA-DT**	172	147	277	312	197, 293, 395
**TPA-tHB**	102	-	393	409	432
**TPA-dHB**	79	-	380	400	439

T_5_, T_10—_temp. based on 5%, 10% weight loss from TGA curves. T_max—_temp. of the maximum decomposition rate from DTG curves. T_m—_melting temp. from the first DSC heating scan. T_g_—glass transition temp. from the second DSC heating scan. - not detected.

**Table 2:** The electrochemical properties (E_HOMO_, E_LUMO_, E_g_) of the investigated azomethines.

Code	Method	E_red_^1^	E_red_^1(onset)^	E_ox_^1^	E_ox_^1(onset)^	E_ox_^2^	E_ox_^3^	E_LUMO_	LUMO ^a^	E_HOMO_	HOMO ^a^	E_g_
		[V]	[V]	[V]	[V]	[V]	[V]	[eV]	[eV]	[eV]	[eV]	[eV]
**TPA-DT**	CV	−2.11	−1.84	0.45	0.26	0.90	nd	−3.26	−2.31	−5.36	−5.07	2.10
	DPV	−2.03	−1.78	0.40	0.20	0.88	nd	−3.32		−5.30		1.98
**TPA-tHB**	CV	nd	nd	0.42	0.29	0.72	1.07	−2.62 ^c^	−2.09	−5.39	−5.34	2.77 ^b^
	DPV	nd	nd	0.38	0.28	0.71	1.02	−2.61 ^c^		−5.38		
**TPA-dHB**	CV	nd	nd	0.53	0.40	0.76	1.22	−2.68 ^c^	−2.13	−5.50	−5.35	2.82 ^b^
	DPV	nd	nd	0.49	0.35	0.70	1.06	−2.63 ^c^		−5.45		

E_HOMO_ = (−5.1-E_ox_^1(onset)^)·|e|, E_LUMO_ = (−5.1-E_red_^1(onset)^)·|e|, E_g_= E_ox_^1(onset)^−E_red_^1(onset)^. Solvent: CH_2_Cl_2_ with c = 10^−3^ mol/dm^3^ and electrolyte 0.1 mol/dm^3^ Bu_4_NPF_6_ and platinum wire as a working electrode. E_ox_ ^1^ the first oxidation process, E_red_^1^ the first reduction process, E_red_ ^1(onset)^ the onset potential of the first reduction process, E_ox_^1(onset)^ the onset potential of the first oxidation process. E_HOMO_ and E_LUMO_ as ionization potential (IP) and electron affinities (EA). V = 0.1 V/s for cyclic voltammetry and v = 0.01 V/s for differential pulse voltammetry. ^a^ LUMO and HOMO calculated by DFT (cf. Section 3.4). nd as not detected. ^b^ E_g_^opt^ is the optical band gap from CH_2_Cl_2_ solution. ^c^ E_LUMO_ = E_HOMO_ + E_g_^opt^.

**Table 3:** UV–vis and PL data of the azomethines.

Code	Medium	UV–Vis	PL		
		λ_max_ (nm), (^b^ ε·10^4^)	λ_em_ (nm)	Stokes Shifts (cm^−1^)	Φ (%)
**TPA-DT**	^a^ C_6_H_5_Cl	287 (0.7), 317 ^sh^ (0.4), 447 (3.1), 471 (3.2)	-	-	-
	^a^ CHCl_3_	321 ^sh^ (1.8), 445 (11), 469 (10.9)	503	2591	2.12
	^a^ CH_2_Cl_2_	250 (1.0), 320 ^sh^ (0.6), 445 (2.7), 466 (2.6)	515	2979	0.36
	Film	317, 455, 483 ^sh^	532	3675	0.22
	imine:PCBM	339, 450 ^sh^	400	4498	-
	imine:P3HT:PCBM	333, 435 ^sh^, 511, 556, 604	380	3714	-
**TPA-tHB**	^a^ C_6_H_5_Cl	288 (1.2), 400 (3.6)	-	-	-
	^a^ CHCl_3_	324 (2.1), 330 ^sh^, 400 (11.4)	454	2974	0.06
	^a^ CH_2_Cl_2_	242 (1.1), 295 (0.6), 336 ^sh^, 398 (3.5)	455	3148	0.50
	Film	339 ^sh^, 398, 422 ^sh^	460	3386	0.84
	imine:PCBM	334, 401	390	4299	-
	imine:P3HT:PCBM	336, 430 ^sh^, 506, 509, 555 ^sh^, 605 ^sh^	393	4316	-
**TPA-dHB**	^a^ C_6_H_5_Cl	289 (1.9), 367 ^sh^ (1.6), 400 (1.1)	-	-	-
	^a^ CHCl_3_	364 ^sh^ (4.6), 401 (6.7), 478 ^sh^ (0.4)	460	3199	0.10
	^a^ CH_2_Cl_2_	248 (0.6), 278 (0.5), 364 ^sh^ (1.2), 401 (2.0)	456	3008	0.04
	Film	370 ^sh^, 400	460	3261	2.22
	imine:PCBM	334, 405	449	3624	-
	imine:P3HT:PCBM	335, 512, 556 ^sh^, 605 ^sh^	388	7668	-
**P3HT**	Donor:PCBM	336, 502, 561 ^sh^, 607 ^sh^	387	3922	-

^a^ c = 10^−5^ moldm^−3^; ^b^ molar absorption coefficients [dm^3^ mol^−1^ cm^−1^], underline data indicates excitation wavelength, ^sh^ shoulder.

**Table 4:** Photovoltaic parameters of fabricated BHJ PV cells.

Active Layer Structure	V_oc_ [mV]	J_sc_ [mA cm^−2^]	FF [–]	PCE [%]	*d* [nm]	RMS [nm]
**TPA-DT**:PC_60_BM (1:1.5)	616 (±10)	0.90 (±0.05)	0.31 (±0.01)	0.18 (±0.02)	40	7
**TPA-tHB**:PC_60_BM (1:1.5)	457 (±4)	0.72 (±0.04)	0.28 (±0.01)	0.09 (±0.03)	45	7
**TPA-dHB**:PC_60_BM (1:1.5)	503 (±15)	0.67 (±0.03)	0.16 (±0.01)	0.05 (±0.02)	55	6
**TPA-DT**:P3HT:PC_60_BM (1:8:13)	416 (±9)	8.20 (±0.13)	0.30 (±0.01)	1.05 (±0.05)	80	5
**TPA-tHB**:P3HT:PC_60_BM (1:8:13)	475 (±12)	1.10 (±0.15)	0.16 (±0.01)	0.15 (±0.02)	85	7
**TPA-dHB**:P3HT:PC_60_BM (1:8:13)	339 (±5)	5.01 (±0.20)	0.30 (±0.01)	0.51 (±0.11)	85	7

## Data Availability

The data presented in this study are available on request from the corresponding author.

## Supplementary Materials

The following supporting information can be downloaded at: https://www.mdpi.com/article/10.3390/ma15207197/s1, Figure S1. NMR Spectra of (a) TPA-DT (b) TPA-tHB and (c) TPA-dHB. Figure S2. Normalized IR Spectra of (a) TPA-DT b) TPA-tHB (c) TPA-dHB. Figure S3. DSC thermograms of (a) TPA-DT, (b) TPA-tHB, (c) TPA-dHB. Figure S4. TGA thermograms of (a) TPA-DT (b) TPA-tHB (c) TPA-dHB. Figure S5. CV scans (a) TPA-DT during oxidation, (b) TPA-DT during reduction, (c) TPA-tBH and (d) TPA-dBH during oxidation processes (0.1 mol/dm^3^ Bu_4_NPF_6_ in CH_2_Cl_2_ with Pt). Figure S6. Calculated geometries of (a) TPA-dHB, (b) TPA-tHB (c) TPA-DT. Figure S7. Contours of HOMO and LUMO of imines. Figure S8. DOS spectra of the imines. Figure S9. Absorption spectra of TPA-DT, TPA-tHB and TPA-dHB in (a) C_6_H_5_Cl and (b) CH_2_Cl_2_. Figure S10. Emission spectra of (a) TPA-DT, (b) TPA-tHB and (c) TPA-dHB in CHCl_3_ and CH_2_Cl_2_. Figure S11. The photocurrent-density voltage curves of tested devices. Table S1. Composition of the selected molecular orbitals of TPA compounds. Table S2. Mean plane angles for TPA molecules. Table S3. Calculated dipole moments [D] in ground and S_1_ states in solvents. Table S4. The calculated electronic transitions corresponding to excitation resulting luminescence in chloroform solution.
