# Supplementary material for: Spectroscopic and Physicochemical Investigations of Azomethines with Triphenylamine Core towards Optoelectronics
Source: Materials (Basel). 2022 Oct 15;15(20):7197. doi: 10.3390/ma15207197 (PMC9612165; doi:10.3390/ma15207197)
Supplement: Supplementary file 1 [file materials-15-07197-s001.zip › materials-1919084-supplementary.pdf]

Spectroscopic and physicochemical investigations of azomethines with triphenylamine core towards optoelectronics

Muhammad Faisal Amin[1], Paweł Gnida[1], Sonia Kotowicz[2], Jan Grzegorz Małecki[2], Mariola Siwy[1], Paweł Nitschke[1], Ewa Schab-Balcerzak [1,2*]

[1]*Centre of Polymer and Carbon Materials, Polish Academy of Sciences, 34 M. Curie-Skłodowska Str., 41-819 Zabrze, Poland*

[2]*Institute of Chemistry, University of Silesia, 9 Szkolna Str., 40-007 Katowice, Poland*

1. Thin films preparation

The solutions in chloroform of the three test compounds were prepared at a concentration of 20 mg/mL. Thin films were created by applying solutions to glass by spin-coating at 1000 rpm. The thin films were then annealed to remove the solvent.

2. Blends preparation

Two types of blends were prepared: two-component and three-component. To obtain the two-component blends, the test compound and the commercial acceptor $PC_{60}BM$ were used in a 1:1.5 ratio. The ternary blends were prepared from a solution containing the test compound, the commercial donor P3HT and the acceptor $PC_{60}BM$ in a ratio of 1:8:13. Solutions were applied to glass substrates using the spin-coating method using 1000 rpm. Reference blends containing P3HT and PCBM in a ratio of 1:1.5 were also prepared. The concentration of the solutions used to prepare the blends was 20 mg/mL as a solvent was used chlorobenzene.

3. Devices preparation

The bulk-heterojuntion solar cells were prepared on the ITO-coated glass (Ossila Ltd. Sheffield. UK. 6 pixels. each with an area of 4.5 mm^2). The ITO substrates were cleaned using isopropanol in the ultrasonic bath. Thin layer of PEDOT:PSS was deposited by spin-coating (5000 rpm) and then annealed at 100 °C for 15 minutes. Solutions of the active layer were prepared by dissolving blends of imines and fullerene derivatives in chlorobenzene using a weight ratio of 1:1.5 or 1:8:13 (imine:P3HT:PCBM). The active layers were deposited by spin-coating at 1000 rpm and subsequently annealed at 120°C for 15 minutes. The aluminum counter electrodes were prepared

by evaporation on the top of active layer. Then the current density-voltage characteristic were registered using PV Test Solutions Solar Simulator under standard conditions (AM 1.5).

a)

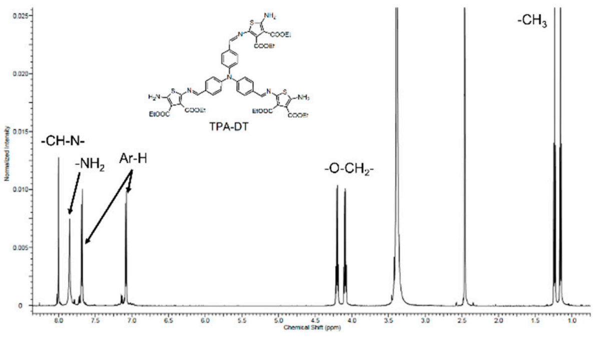

b)

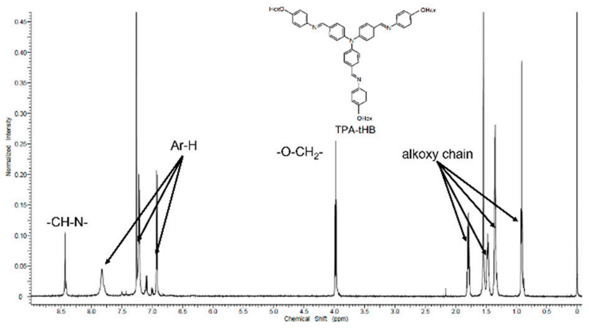

c)

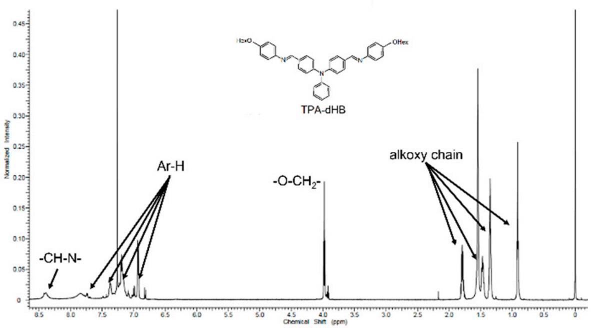

Figure S1. NMR Spectra of a) **TPA-DT** b) **TPA-tHB** and c) **TPA-dHB**

a)

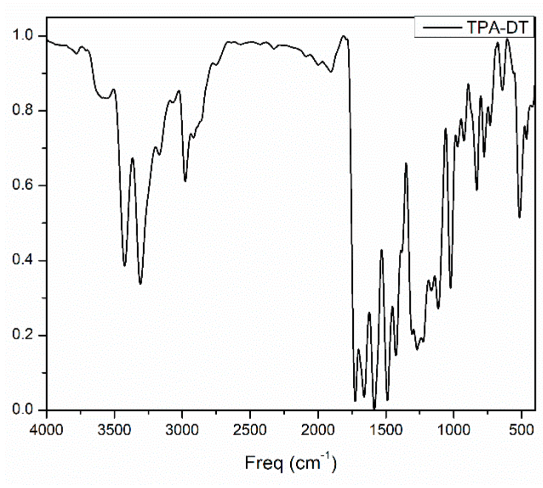

b)

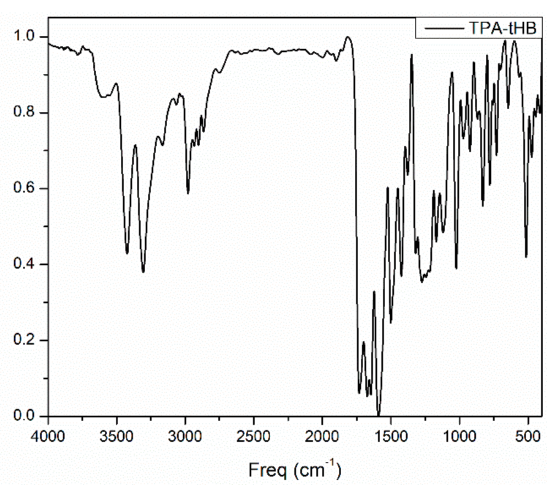

c)

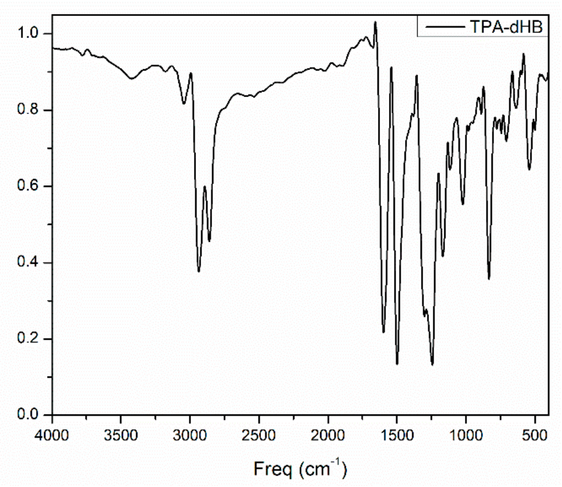

Figure S2. Normalized IR Spectra of a) **TPA-DT** b) **TPA-tHB** c) **TPA-dHB**

a)

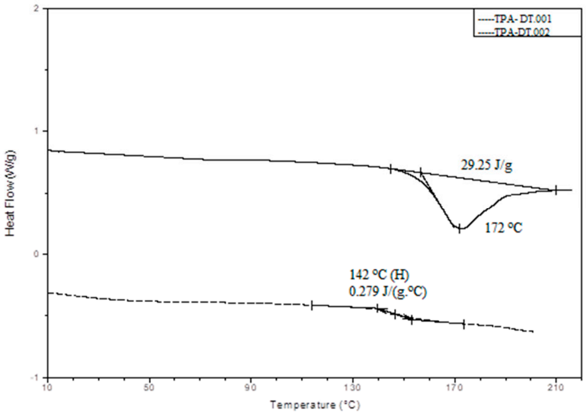

b)

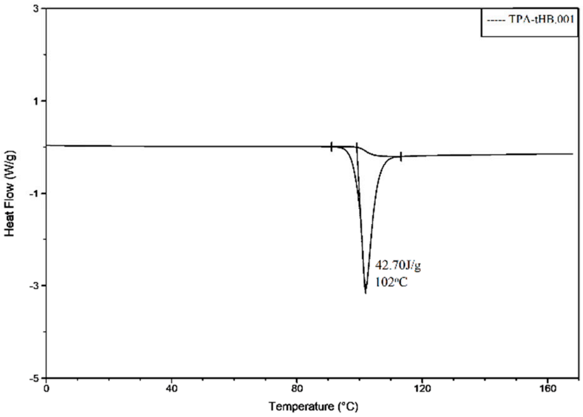

c)

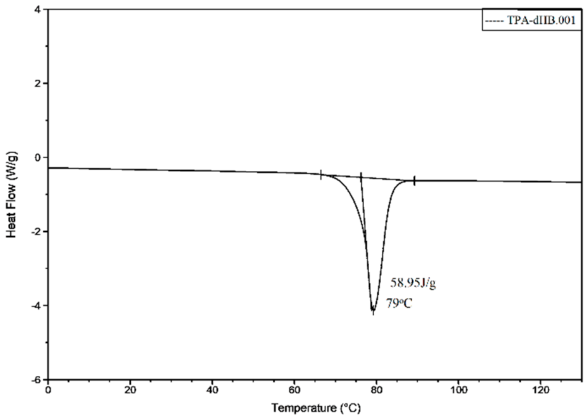

Figure S3. DSC thermograms of a) **TPA-DT**, b) **TPA-tHB**, c) **TPA-dHB**

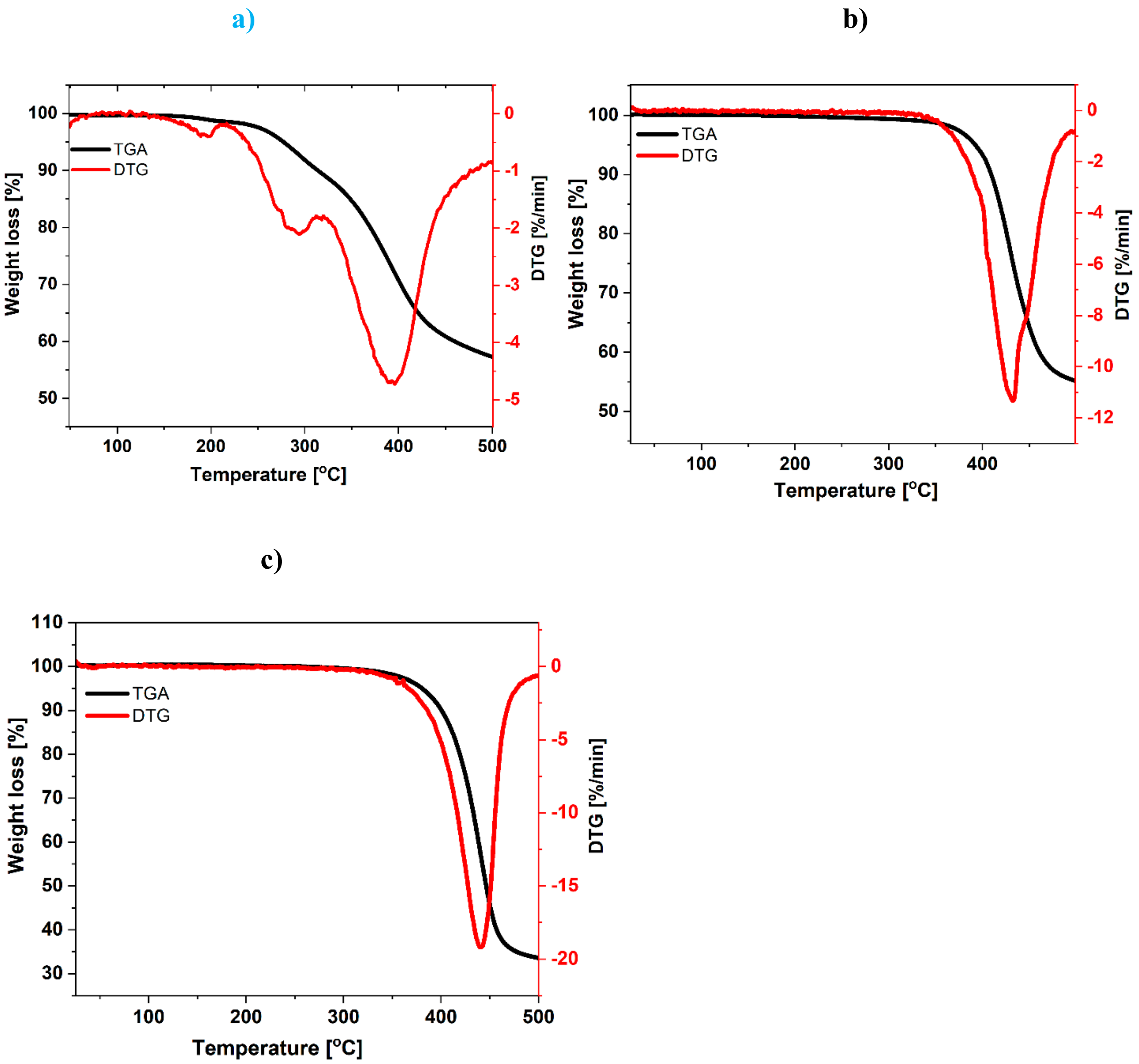

Figure S4. TGA thermograms of a) **TPA-DT** b) **TPA-tHB** c) **TPA-dHB**.

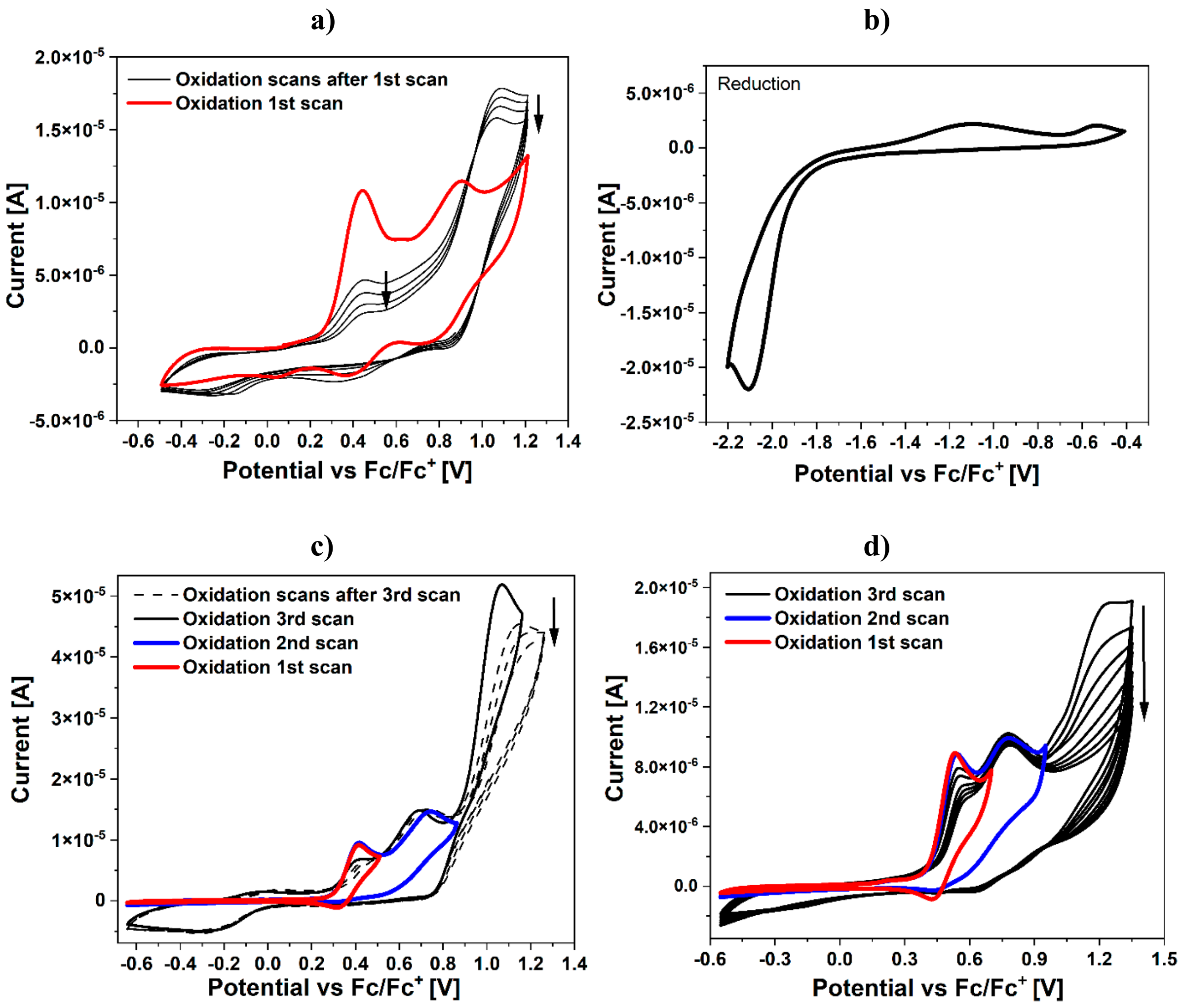

Figure S5. CV scans a) **TPA-DT** during oxidation, b) **TPA-DT** during reduction, c) **TPA-tBH** and d) **TPA-dBH** during oxidation processes (0.1mol/dm^3 Bu_4NPF_6 in CH_2Cl_2 with Pt).

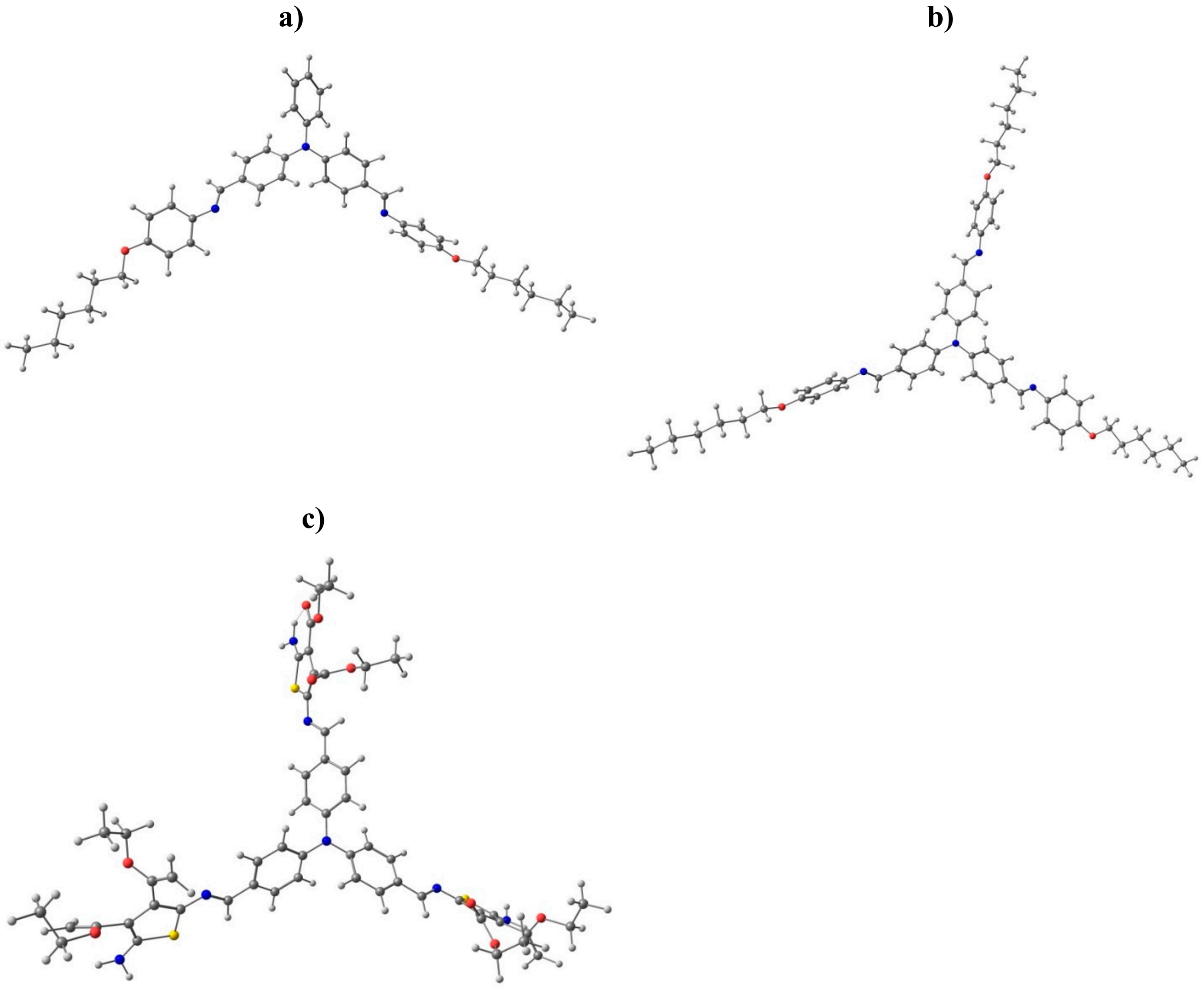

Figure S6. Calculated geometries of a) **TPA-dHB**, b) **TPA-tHB** c) **TPA-DT**.

HOMO	LUMO
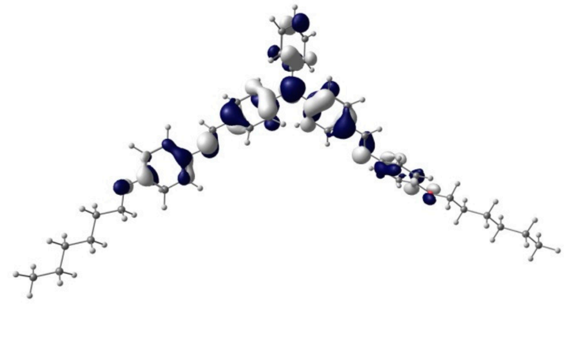	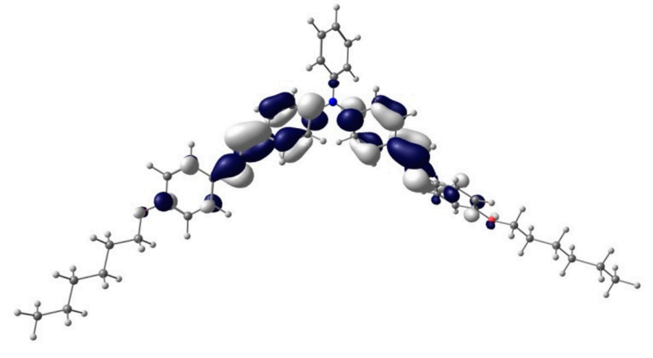
TPA-dHB	
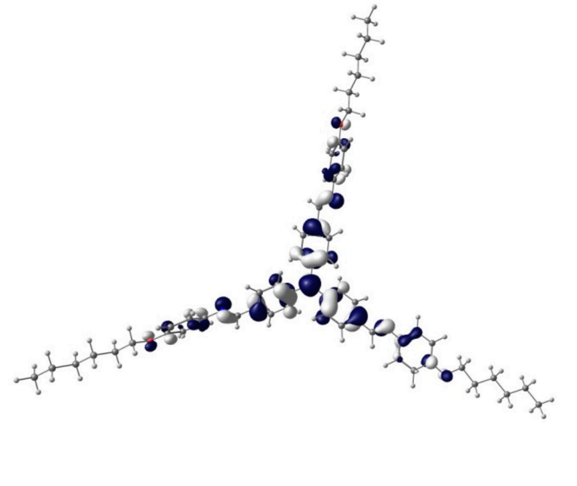	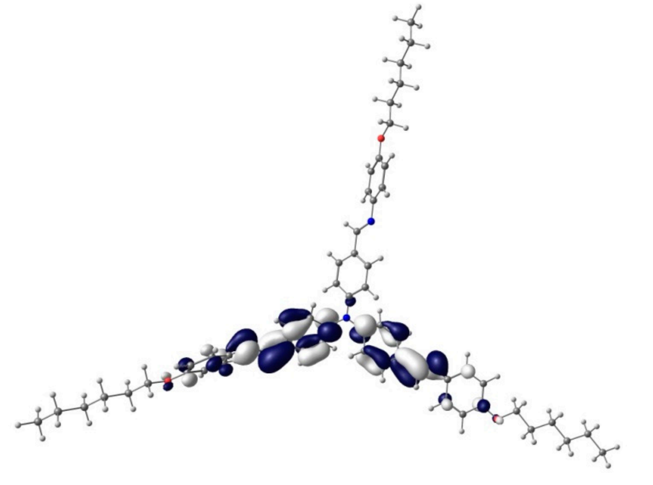
TPA-tHB	
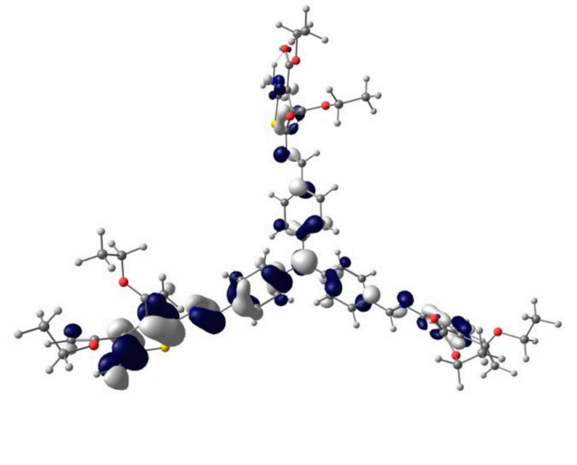	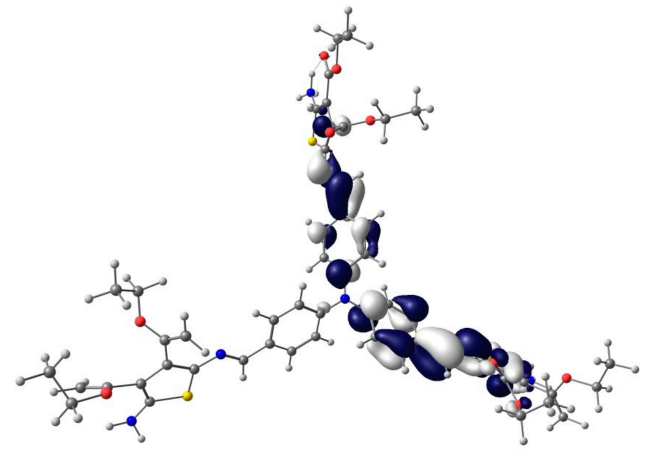
TPA-DT	

Figure S7. Contours of HOMO and LUMO of imines.

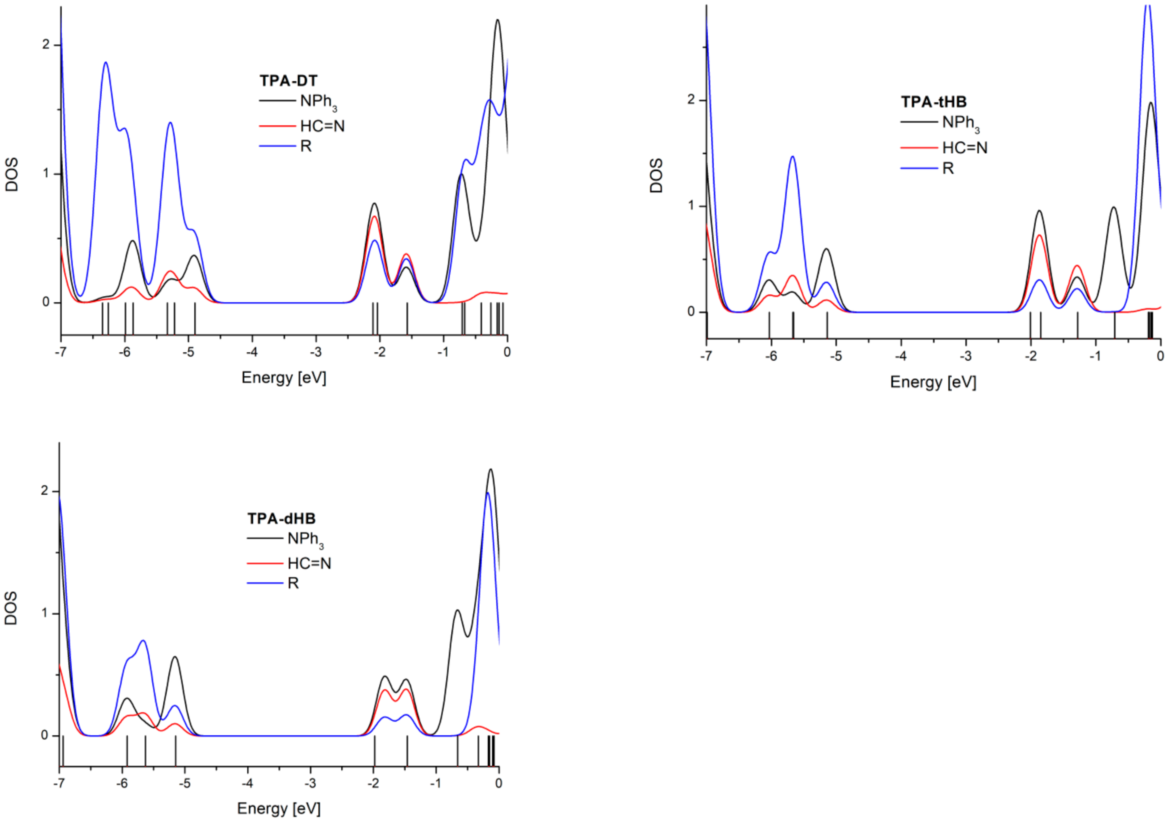

Figure S8. DOS spectra of the imines.

a) **b)**

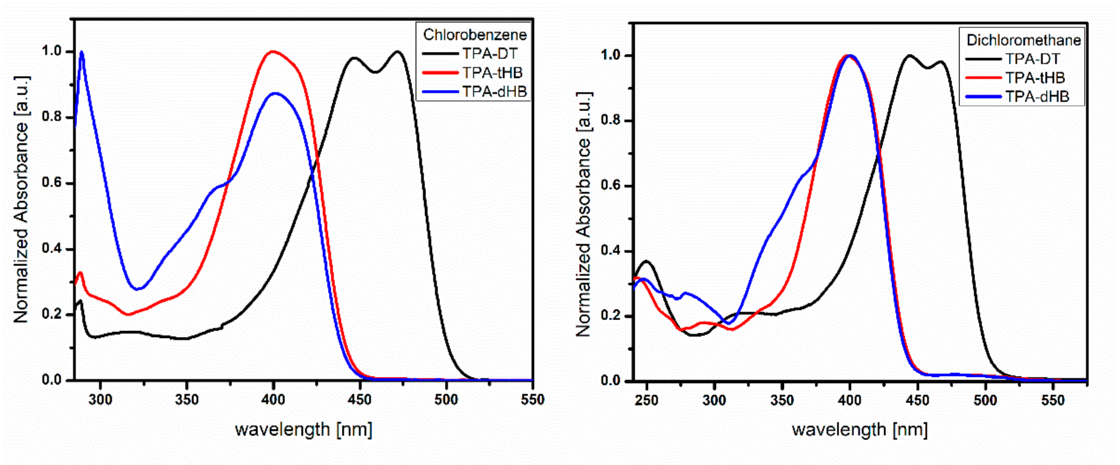

Figure S9. Absorption spectra of **TPA-DT**, **TPA-tHB** and **TPA-dHB** in a) C_6H_5Cl and b) CH_2Cl_2.

a) **b)**

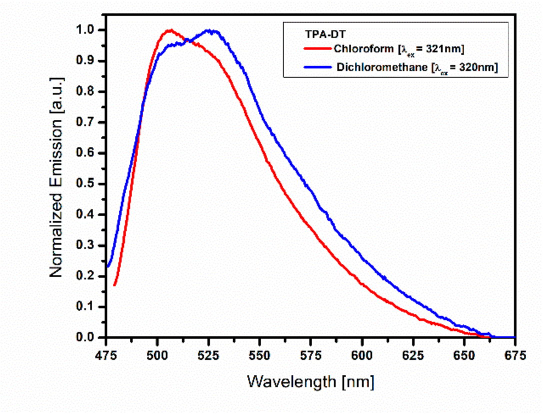

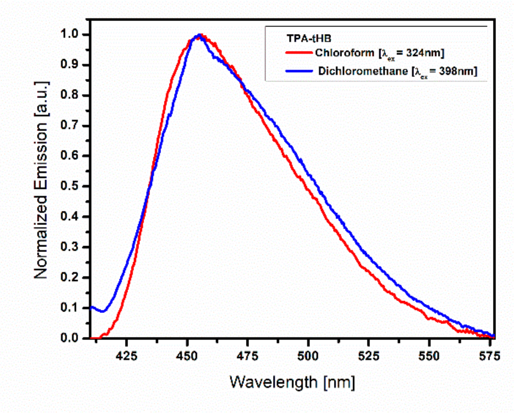

c)

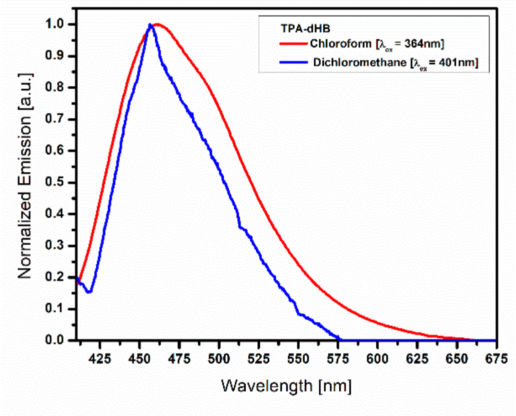

Figure S10. Emission spectra of a) **TPA-DT**, b) **TPA-tHB** and c) **TPA-dHB** in $CHCl_3$ and CH_2Cl_2.

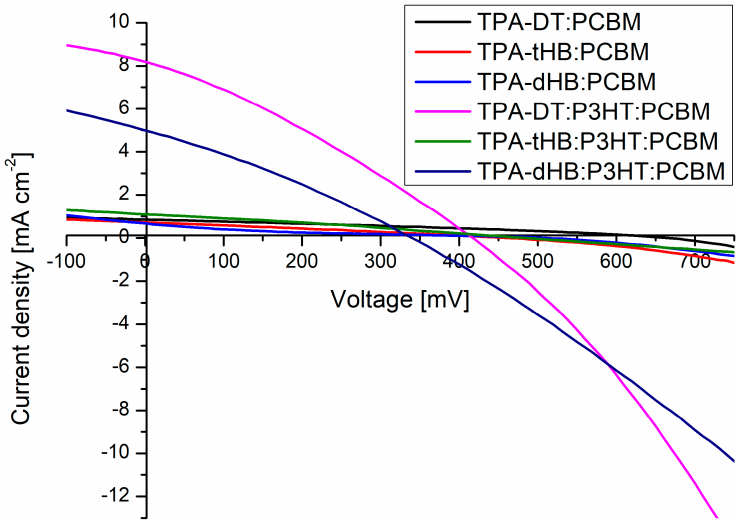

Figure S11. The photocurrent-density voltage curves of tested devices.

Table S1. Composition of the selected molecular orbitals of TPA compounds.

TPA-dHB	eV	NPh_3	HC=N	R	TPA-tHB	eV	NPh_3	HC=N	R
L+5	-0.19	7	1	92	L+5	-0.22	2	1	97
L+4	-0.21	1	1	98	L+4	-0.22	1	1	98
L+3	-0.34	79	6	15	L+3	-0.73	99	0	0
L+2	-0.67	100	0	0	L+2	-1.31	33	44	23
L+1	-1.51	44	38	18	L+1	-1.88	47	37	16
LUMO	-2.13	47	37	16	LUMO	-2.09	47	37	16
HOMO	-5.35	67	10	23	HOMO	-5.34	62	12	27
H-1	-5.65	10	17	73	H-1	-5.67	10	17	73
H-2	-5.93	29	14	57	H-2	-5.68	10	17	73
H-3	-6.95	70	18	12	H-3	-6.05	29	15	56
H-4	-7.00	3	5	92	H-4	-6.99	45	21	34
H-5	-7.04	22	3	74	H-5	-7.02	26	18	56

TPA-DT	eV	NPh_3	HC=N	R
L+5	-0.63	30	6	63
L+4	-0.87	97	0	2
L+3	-1.01	1	0	99
L+2	-1.77	26	37	37
L+1	-2.24	38	34	28
LUMO	-2.31	38	35	27
HOMO	-5.07	38	11	51
H-1	-5.39	12	13	75
H-2	-5.50	10	14	76
H-3	-6.05	46	10	44
H-4	-6.19	1	3	96
H-5	-6.45	3	2	95

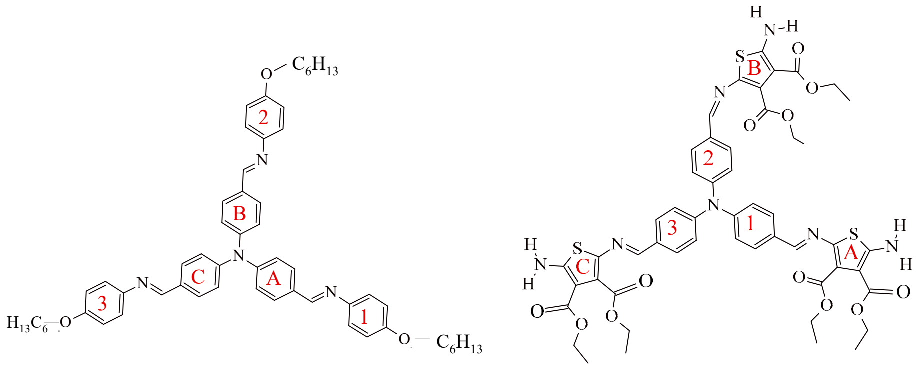

Table S2. Mean plane angles for TPA molecules.

compound	state	∡AB [°]	∡BC [°]	∡CA [°]	∡A1 [°]	∡B2 [°]	∡C3 [°]
TPA-DT	S_0	68.55	68.15	69.65	4.88	35.85	42.42
	S_1	55.58	78.29	89.19	35.05	10.09	35.03
TPA-tHB	S_0	66.38	66.19	66.89	33.06	32.82	35.08
	S_1	67.58	67.51	59.37	22.71	23.37	23.03
TPA-dHB	S_0	71.05	71.22	65.12	81.68		34.94
	S_1	64.76	67.96	63.09	20.22		23.07

Table S3. Calculated dipole moments [D] in ground and S_1 states in solvents.

	TPA-DT	TPA-dHB	TPA-tHB
S_0			
C_6H_5Cl	5.69	2.89	1.52
$CHCl_3$	5.60	2.53	1.49
NMP	6.19	3.30	1.63
S_1			
C_6H_5Cl		2.99	1.12
$CHCl_3$		2.91	1.10
NMP		3.40	1.22

Table S4. The calculated electronic transitions corresponding to excitation resulting luminescence in chloroform solution.

	λ_{exp}	λ_{calc} (*f*)	Transition	Character
TPA-DT	367	374.0 (0.1031)<br>369.7 (0.0885)	H-1→L+2 (96%)<br>H-3→LUMO (94%)	$\pi_R \rightarrow \pi^*$<br>$\pi_{R/NPh3} \rightarrow \pi^*$
TPA-tHB	353	352.7 (0.0093)	HOMO→L+2 (90%)	$\pi_{R/NPh3} \rightarrow \pi^*$
TPA-dHB	376	381.0 (0.6454)	HOMO→L+1 (96%)	$\pi_{R/NPh3} \rightarrow \pi^*_{NPh3/HC=N}$
